# Genome-Wide Association Study of Abdominal and Intramuscular Fat Deposition Traits in Huainan Yellow-Feathered Chickens

**DOI:** 10.3390/ani15223342

**Published:** 2025-11-19

**Authors:** Zichun Dai, Yaxin Li, Jie Liu, Rong Chen, Huanxi Zhu, Mingming Lei

**Affiliations:** 1 Institute of Animal Science, Jiangsu Academy of Agricultural Sciences, Nanjing 210014, China; 20210064@jaas.ac.cn (Z.D.); liyaxin6664@163.com (Y.L.); liujie891213@163.com (J.L.); chenrong_big@163.com (R.C.); xuanzaizhu@163.com (H.Z.); 2 Key Laboratory of Crop and Livestock Integration, Ministry of Agriculture, Nanjing 210014, China; 3 Jiangsu Province Engineering Research Center of Precision Animal Breeding, Nanjing 210014, China

**Keywords:** Huainan yellow chicken, abdominal fat deposition, intramuscular fat, GWAS, candidate genes

## Abstract

The Huainan yellow-feathered chicken is a prized local breed known for its high-quality meat. However, excessive abdominal fat deposition adversely affects feed efficiency and carcass quality. This study aimed to identify genetic markers and candidate genes associated with fat traits to facilitate marker-assisted selection (MAS) using genome-wide association studies (GWAS). A total of 220 chickens were phenotyped for abdominal fat weight (AFW), abdominal fat percentage (AFP), intramuscular fat of pectoral muscle (IFPM), and intramuscular fat of leg muscle (IFLM). GWAS based on whole-genome resequencing revealed significant SNPs for AFW and AFP on chromosomes 1, 2, 7, 10, 13, and 35, annotating genes including *GRIA1*, *CYP1A1*, *CYP1A2*, and *SCAMP2*. For IFPM and IFLM, significant loci were identified on chromosomes 1, 2, 4, 5, 6, 9, 12, 23, 25, 26, and 28, highlighting genes such as *LRP4*, *FABP3*, and *ADAMTS9*. Functional enrichment analysis showed involvement of steroid hormone biosynthesis, retinol metabolism, and cytochrome P450 pathways in abdominal fat deposition, while Wnt and MAPK signaling pathways regulated intramuscular fat. These findings provide molecular targets for genetic selection to improve fat traits in Huainan chickens.

## Simple Summary

The Huainan yellow-feathered chicken is a prized local breed known for its high-quality meat. However, excessive abdominal fat deposition adversely affects feed efficiency and carcass quality. Since this fat is difficult to measure in live birds, breeding for leaner chickens is challenging. In this study, we used a genetic approach to identify DNA markers linked to fat deposition. We discovered several key genes that control processes like appetite, fat breakdown, and cholesterol transport. Our findings provide new tools for breeders to select chickens that are genetically inclined to be leaner, which will help improve meat quality and reduce waste fat in this prized local breed.

## 1. Introduction

Chinese yellow-feathered broilers are widely recognized for their high-quality meat and represent an important source of premium animal protein. Although long-term systematic breeding has substantially enhanced their meat production performance, excessive abdominal fat deposition has become an increasingly prominent issue [1]. This not only reduces feed efficiency and increases rearing costs, but may also compromise carcass yield, meat quality attributes, and consumer health [2]. Currently, abdominal fat traits cannot be measured directly in live animals, and accurate assessment relies solely on post-slaughter dissection—a process that is costly, time-consuming, and labor-intensive. These limitations severely impede the genetic improvement of abdominal fat traits through phenotypic selection [3].

Fat deposition is influenced by both genetic and nutritional factors, including an individual’s genetic background, genomic polymorphisms, and gene expression patterns [4]. Carcass traits generally exhibit moderate to high heritability, though this varies across populations and specific traits [5]. Previous studies have indicated that abdominal fat traits in chickens are highly heritable, with an estimated heritability of approximately 0.82, highlighting substantial potential for genetic improvement [6]. Therefore, identifying molecular markers associated with abdominal fat deposition, elucidating the underlying genetic mechanisms, and implementing marker-assisted selection (MAS) are of great importance for reducing abdominal fat content and enhancing breeding efficiency in Huainan yellow chickens.

Genome-wide association studies (GWAS) offer a high-throughput strategy for systematically identifying single nucleotide polymorphisms (SNPs) significantly associated with target traits across the genome. Since the pioneering GWAS on age-related macular degeneration by Klein et al. in 2005 [7], this approach has been extensively applied in genetic analyses and molecular breeding for economically important traits. In poultry, multiple SNP loci and candidate genes related to fat deposition—particularly abdominal fat—have been identified in both white-feathered broilers and Chinese indigenous yellow chickens [8]. SNPs can influence phenotypic variation by interacting with transcription factors and regulating the expression of key genes involved in adipocyte differentiation and proliferation [9]. These findings underscore the importance of clarifying the genetic basis of fat deposition and discovering potential candidate genes.

In this study, we performed a GWAS using whole-genome resequencing to investigate fat-related traits in Huainan yellow chickens. Our objective was to identify significant SNPs and key genes associated with abdominal fat deposition, with the aim of providing novel genetic markers for marker-assisted selection and laying a theoretical foundation for genetic improvement of fat traits in this breed.

## 2. Materials and Methods

### 2.1. Ethics Approval

The experimental protocol was approved by the Research Committee of the Jiangsu Academy of Agricultural Sciences and was conducted in compliance with the Regulations for the Administration of Affairs Concerning Experimental Animals (Decree No. 63), issued by the Jiangsu Academy of Agricultural Sciences on 8 July 2014.

### 2.2. Population and Experimental Design

The experiment was conducted at the Liuhe Experimental Poultry Farm of the Jiangsu Academy of Agricultural Sciences in Nanjing, China. A total of 220 healthy 35-day-old Huainan yellow chickens (a local breed from Anhui Province), comprising 100 roosters and 120 hens—were used in this study. These birds (100 roosters and 120 hens) were randomly selected from a larger population to ensure a representative sample for the study. All birds were raised under environmentally controlled conditions (temperature maintained at 24 °C; relative humidity 85–90%) using an online flat-feeding system. They were provided with ad libitum access to water and fed a standard growth pellet diet containing 20% crude protein and 12.54 MJ/kg metabolizable energy. The detailed composition and nutritional content of the diet provided to the chickens are summarized in Supplementary Table S1. Uniform immunization was performed following the routine broiler vaccination protocol.

Slaughter and sampling were carried out when the chickens reached 110 days of age. After a 24 h fasting period, blood samples were collected from the inferior vena cava using EDTA-coated tubes for anticoagulation. Birds were stunned using an electric shock (120 V, 50 Hz, 5 s) prior to exsanguination, which was performed by severing the jugular vein and carotid artery on one side of the neck. After slaughter, feathers, head, internal organs, and claws were removed, and the remaining carcass, including abdominal fat, was weighed to obtain the carcass weight. Abdominal adipose tissue and other extragastric fat deposits were carefully separated and weighed. The heart and liver were also excised and weighed accordingly.

### 2.3. Statistical Analysis

#### 2.3.1. Phenotypic Data Statistical Analysis

Carcass weight (CW), liver weight (LVW), abdominal fat (AFW), and heart weight (HEW) were calculated using Microsoft Excel. The indices of liver (LVR), abdominal fat (AFP), and heart (HER) were calculated as follows:

Liver or abdominal fat or heart index = [tissue weight g/carcass weight g] × 100%.

Descriptive statistical analysis was conducted on the abdominal fat weight and abdominal fat percentage of Huainan yellow chickens using SPSS software (SPSS 29.0, SPSS Inc., Chicago, IL, USA). The sample size (N), maximum value (Max), minimum value (Min), mean value (Mean), and standard deviation (SD) were calculated.

Analysis of correlation was performed using SPSS Statistics (SPSS 29.0, SPSS Inc., Chicago, IL, USA). Normality of the data distribution for all continuous variables was assessed using the Shapiro–Wilk test and by visual inspection of Q-Q plots. The association between continuous variables was evaluated using bivariate correlation analysis. Correlation coefficients (r) were interpreted as follows: |r| < 0.3 indicated a weak correlation, 0.3 ≤ |r| < 0.5 indicated a moderate correlation, and |r| ≥ 0.5 indicated a strong correlation.

#### 2.3.2. DNA Extraction and Low Depth Sequencing

Genomic DNA was extracted from blood samples with a high-throughput DNA extraction kit, and its purity and integrity were detected by 1.5% agarose gel electrophoresis. For qualified DNA samples, Tn5 transposase was used for random disruption, followed by PCR amplification and magnetic bead sorting to construct a sequencing library. The insertion fragment size of the library was controlled between 300–600 bp. Subsequently, low-depth whole genome resequencing was performed on the DNBSEQ-T7 platform, with an average sequencing depth of 10× across all samples. Perform quality control and sequence alignment on the raw sequencing data to obtain high-quality and effective data, resulting in a final dataset with a mean mapping rate of >95% to the reference genome (GRCg7b).

#### 2.3.3. Genotyping Data Processing and Population Structure Analysis

Low-quality sequencing data were filtered using Trimomatic software (v0.39), and high-quality sequences were aligned to the reference genome of domestic chickens (GRCg7b) using BWA software (version 0.7.17), which provides a high-quality and widely used genomic framework for variant discovery in domestic chickens. SNP detection was performed using GATK V4.1.8.1 software, and strict quality control was carried out according to the following criteria: loci with Quality by Depth (QD) < 2.0, Mapping Quality (MQ) < 40.0, or Fisher Strand (FS) > 60.0 were excluded. After filtering, a total of 514,882 high-quality SNPs were retained for subsequent analysis. To eliminate the interference of linkage disequilibrium on analysis, independent SNPs were screened using PLINK software (v1.90), and principal component analysis (PCA) was performed based on this to evaluate population structure.

#### 2.3.4. GWAS of Four Fat Traits

Based on a high-quality SNP dataset, a genome-wide association study was performed for each fat trait using the Fixed and Random Model Circulating Probability Unification (FarmCPU) model, as implemented in the R package GAPIT3 Version 3. The model is as follows:

Y = Xβ + Sα + Zu + e

where Y is the phenotype values, X and Z are the design matrices, S is the SNP genotype matrix, β is the fixed effect vector for environments and sex, α is effect vector for the SNP marker genotype, u is the random effect caused by polygenic background, and e is the residual effect. The Bonferroni correction method was used to stringently adjust for multiple tests to control false positive rates. The significance threshold at the genomic level was set to *p* < 0.01/N, and the suggestive association threshold was set to *p* < 0.05/N, where N is the number of independent SNPs used for analysis.

#### 2.3.5. Candidate Genes Annotation and Functional Enrichment Analysis

A 1% threshold was applied in accordance with previous literature and the expected average proportion of genetic variance explained by a single chromosome segment. For each window, the most significant SNP was selected as the midpoint, and the region was extended by 0.1 Mb in both upstream and downstream directions. These expanded regions were subsequently used for candidate gene screening. Candidate genes within the identified QTL regions were retrieved from the Ensembl database (*Gallus_gallus*-7.0; https://asia.ensembl.org, accessed on 10 July 2025). Kyoto Encyclopedia of Genes and Genomes (KEGG) enrichment analyses were conducted based on the candidate genes using Metascape v2.2.2.20723 [10] for functional annotation, visualization, and integrated discovery.

## 3. Results and Discussion

### 3.1. Statistical Data of Fat Traits and Fat-Related Traits

Descriptive statistics for nine fat and fat-related traits in Huainan yellow-feathered chickens are summarized in Table 1. The mean values of the traits were as follows: CW (1615.27), LVW (35.59), AFW (53.79), HEW (8.99), LVR (2.26), AFP (3.18), HER (0.56), intramuscular fat of pectoral muscles (IFPM) (4.19), and intramuscular fat of leg muscles IFLM (7.48). The coefficients of phenotypic variation (CV) ranged from 19.15% (HER) to 51.82% (AFW), with AFW and AFP showing the highest coefficients of variation. This suggests that heart development is relatively stable, whereas abdominal fat deposition exhibits substantial variability, reflecting considerable genetic diversity across the measured traits. The CV for both AFW and AFP in Huainan yellow chickens was high, which is consistent with Pan’s findings in dwarf yellow-plumage chickens [8]. The result indicated that genomic-assisted selection is a promising strategy to improve fat traits in breeding programs.

To further explore the relationships among these traits, we performed a correlation analysis (Table 2). AFP showed significant positive correlations with CW (r = 0.264, *p* < 0.01) and IFLM (0.275, *p* < 0.01), and significant negative correlations with LVW (r = −0.167, *p* < 0.05), LVR (r = −0.329, *p* < 0.01), and HER (r = −0.345, *p* < 0.01). IFPM was positively correlated with LVW (r = 0.169, *p* < 0.05), and LVR (r = 0.242, *p* < 0.01). IFLM demonstrated relatively strong phenotypic associations with AFW (r = 0.276, *p* < 0.01) and AFP (r = 0.275, *p* < 0.01), whereas correlations between abdominal fat traits and pectoral muscle fat traits were weak. In addition, CW was significantly correlated with HEW (r = 0.701, *p* < 0.01), LVW was significantly correlated with LVR (r = 0.743, *p* < 0.01), HEW was significantly correlated with HER (r = 0.875, *p* < 0.01), AFW was significantly correlated with AFP, with a correlation coefficient of up to 0.989 (*p* < 0.01).

### 3.2. GWAS for AFW and AFP

As important quantitative traits in poultry, abdominal fat deposition plays a critical role in assessing chicken production performance. To pinpoint potential candidate genes, we first identified key genomic regions. Specifically, these regions were defined by extending 100 kb upstream and downstream from each GWAS significant SNP and then intersecting the intervals with protein-coding gene annotations.

**Figure 1:**
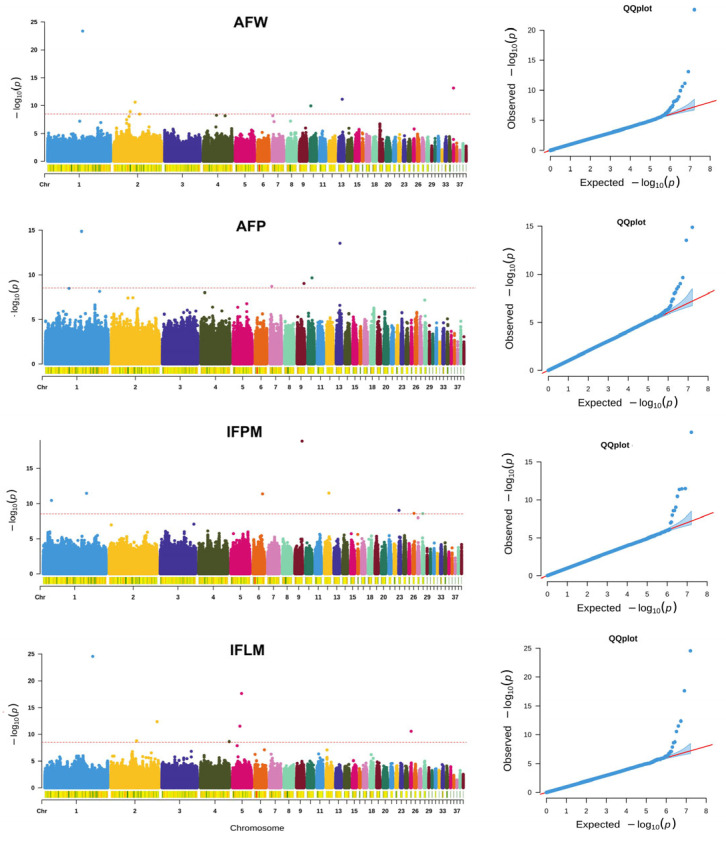
Proportion of genetic variances of four fat traits explained by 0.05 Mb windows. AFW: abdominal fat weight; AFP abdominal fat percentage; IFPM: intramuscular fat of pectoral muscle; IFLM: intramuscular fat of leg muscle.

For AFW, six significant SNPs (*p* < 1 × 10^−9^) were detected on *Gallus gallus* chromosomes (GGC) 1, 2, 10, 13, and 35, with genetic effects ranging from −12.54 to 30.32. A total of 28 genes were annotated within these associated regions (Figure 1; Table 3). For abdominal fat percentage (AFP), five significant regions were identified on GGC 1, 7, 9, 10, and 13, with effect sizes between −1.75 and 1.93, encompassing 16 candidate genes.

Notably, due to the strong phenotypic correlation between AFW and AFP, a shared significant SNP (rs11945109, T/A) was identified on GGC13. This variant was found within an intron of the *glutamate ionotropic receptor AMPA type subunit 1* (*GRIA1*) gene, a gene spanning the 11.92–12.04 Mb region on GGC13, at position 11,945,109. The *GRIA1*-encoded GluA1 protein is a core component of the AMPA receptor and has been implicated in central regulation of appetite and energy balance [11,12], suggesting its potential role in modulating feed intake and fat deposition. Supporting this, studies in mice have shown that high-fat diet feeding significantly upregulates hypothalamic expression of *AMPA receptor subunits*, including *GRIA1* [13]. These findings collectively nominate *GRIA1* as a promising candidate gene influencing both AFW and AFP.

Another SNP, rs66761782 on GGC13, was localized within a region containing 16 genes, several of which have established roles in lipid metabolism. Among these, cytochrome P450 family members *CYP1A1*, *CYP1A2*, and *CYP11A1* are involved in steroidogenesis and xenobiotic metabolism. *CYP1A1* promotes preadipocyte maturation [14,15], *CYP11A1* initiates steroid hormone synthesis by converting cholesterol to pregnenolone [16], and *CYP1A2* polymorphisms modulate metabolic responses to dietary factors and obesity susceptibility [17,18]. Other relevant genes in this region include *secretory carrier membrane protein 2* (S*CAMP2*), which participates in cellular cholesterol efflux in foam cells [19,20]; *nudix motif 3* (N*UDT3*) gene negatively regulates adipogenesis and lipid storage [21,22]; and *SH3-domain binding protein 4* (*SH3BP4*) inhibits adipocyte differentiation via mTORC1 suppression [23].

We also identified *UDP-glucuronosyltransferase 1 A1* (*UGT1A1*), which encodes the key enzyme in bilirubin conjugation. Bilirubin, a heme catabolite, has been inversely associated with obesity in both clinical and animal studies [24]. It functions as an activator of peroxisome proliferator activated receptor α (PPARα), promoting fatty acid oxidation and reducing hepatic lipid accumulation [25]. These mechanisms support the notion that bilirubin metabolism may influence obesity and related metabolic disorders.

### 3.3. GWAS for IFPM and IFLM

For IFPM, genome-wide association analysis identified eight significant SNPs distributed across *Gallus gallus* chromosomes (GGC) 1, 6, 9, 12, 23, 26, and 28. These SNPs showed genetic effects ranging from 0.24 to 1.37, and a total of 44 genes were annotated within the corresponding genomic intervals (Figure 1; Table 3). For IFLM, seven significant regions were detected on GGC 1, 2, 4, 5, and 25, with effect sizes between –1.31 and 2.76, encompassing 41 candidate genes.

Several promising candidate genes located within these regions have established roles in lipid metabolism and adipogenesis. *Low-density lipoprotein receptor-related protein 4 (LRP4)*, which encodes a membrane-bound receptor, modulates the uptake and storage of triglyceride-rich lipoproteins in adipose tissue [26,27]. Adipose-specific knockout of *LRP4* in mice reduces fat mass, decreases body weight, and improves systemic glucose and lipid homeostasis [28]. *RAD51B*, a member of the RAD51 protein family, has been associated with body mass index and obesity risk in human genetic research [29]. *Pygo2* functions as a key component of the Wnt/β-catenin signaling pathway and inhibits adipogenesis by suppressing master regulators such as C/EBPα and PPARγ, thereby influencing susceptibility to obesity and diabetes [30].

*Fatty acid-binding protein 3 (FABP3)*, highly expressed in muscle tissues, facilitates the intracellular transport of long-chain fatty acids to mitochondria for β-oxidation or to the endoplasmic reticulum for esterification and storage [31,32]. This process supports energy production in muscle and limits ectopic lipid accumulation [33,34]. *Metalloproteinase with thrombospondin 9A* (*DAMTS9*), which encodes an extracellular protease involved in ECM remodeling, modulates the tissue microenvironment and has been associated in genome-wide studies with systemic obesity and fat distribution [35,36,37]. Additionally, *four and a half LIM domains protein 2* (*FHL2*), which encodes a transcriptional cofactor, expressed in muscle, appears to suppress adipogenic differentiation of mesenchymal stem cells [38,39]. Consistent with this, *FHL2*-deficient mice are resistant to high-fat diet-induced weight gain and maintain glucose homeostasis [40].

### 3.4. KEGG Analysis

KEGG enrichment analysis revealed several significantly enriched pathways associated with the identified candidate genes (Table 4). For the AFW trait, nine signaling pathways were significantly enriched. Among them, the steroid hormone biosynthesis and ovarian steroidogenesis pathways suggest an important role of steroid hormone biosynthesis in abdominal fat development in broilers [41]. The enrichment of multiple additional pathways was observed. Among them, caffeine, linoleic acid, retinol, tryptophan, and xenobiotic/drug metabolism by cytochrome P450, along with chemical carcinogenesis (DNA adducts), all functionally converged on *CYP1A1* and *CYP1A2*. This pattern underscores the central role of the cytochrome P450 system in these metabolic networks. Notably, caffeine metabolism is predominantly mediated by *CYP1A2* [42]. Moreover, bioactive metabolites derived from these pathways—such as retinoic acid, epoxyeicosatrienoic acids (EETs), and tryptophan derivatives—often serve as ligands for nuclear or membrane receptors. This interaction creates a regulatory feedback that influences both cytochrome P450 expression and overall lipid deposition [43,44]. AFW and AFP shared one common pathway—retinol metabolism—whose active metabolite, retinoic acid, is a known suppressor of preadipocyte differentiation [45].

For the IFPM trait, six pathways were enriched, including the MAPK signaling pathway, Wnt signaling pathway, phospholipase D signaling pathway, endocytosis, Fc gamma R-mediated phagocytosis, and choline metabolism in cancer. In contrast, for IFLM, three pathways were significantly enriched: other glycan degradation, sphingolipid metabolism, and terpenoid backbone biosynthesis.

Several of these pathways have established roles in muscle and fat biology. The Wnt/β-catenin pathway promotes myoblast differentiation while inhibiting adipogenesis [46,47]. The phospholipase D pathway participates in diverse cellular processes, including signal transduction, lipid droplet formation, and apoptosis. Phospholipid metabolism also influences meat quality attributes such as texture, flavor, and nutritional value [48,49]. Sphingolipids, including ceramides, can induce insulin resistance and impair glucose uptake in muscle cells [50], or modulate fatty acid β-oxidation [51], thereby affecting energy balance and lipid storage. The enrichment of the glucosylceramidase gene (LOC107050229) within the sphingolipid metabolism pathway further suggests its potential role in regulating intramuscular fat deposition in chickens. Although abdominal fat and intramuscular fat were driven by different key genes and enriched in different upstream metabolic pathways, they shared core cellular signaling pathways such as Wnt and MAPK. These pathways may serve as integration points, synergistically regulating the overall deposition and distribution of body fat through differences in activity in different tissues.

This study has limitations including a modest sample size and single-breed design, which may restrict power to detect minor variants and generalizability. Candidate gene functions remain computationally inferred without experimental validation. Future work should integrate transcriptomic, epigenomic, or metabolomics data to verify causative mechanisms and broaden applicability across breeds. Once validated, these SNPs can serve as molecular markers for the precision breeding of Huainan yellow chickens, enabling the early selection of ideal fat traits. This process is not only expected to improve feed efficiency by reducing abdominal fat deposition, but may also increase intramuscular fat (IMF) content, resulting in excellent meat quality and significantly accelerating genetic processes.

## 4. Conclusions

This study represents the comprehensive GWAS of fat deposition traits in Huainan yellow chickens utilizing whole-genome resequencing. We have successfully identified multiple significant SNPs and candidate genes associated with fat traits in Huainan yellow chickens (e.g., *GRIA1*, *CYP1A1/2*, *LRP4*, *FABP3*), as well as key biological pathways (such as steroid hormone biosynthesis and Wnt signaling). These findings provide valuable molecular markers and theoretical support for the genetic improvement of fat-related traits. By utilizing this genetic information, individuals with low abdominal fat and high intramuscular fat content can be selected to improve feed efficiency, carcass quality, shorten generation intervals, and enhance breeding efficiency, providing valuable resources for future research on the genetic basis of fat traits.

## Figures and Tables

**Table 1:** Descriptive statistics of abdominal fat percentage in Huainan Yellow chickens.

Traits ^a^	Nb	Max	Min	Mean	SD	CV (%)
CW	211	2606	860	1615.27	291.95	18.07
LVW	211	84.02	21.88	35.59	9.33	26.22
AFW	211	122.9	14.18	53.79	27.88	51.82
HEW	211	16.79	4.65	8.99	2.38	26.51
LVR	211	6.26	1.37	2.26	0.68	30.07
AFP	211	0.84	6.4	3.18	1.46	45.89
HER	211	0.96	0.32	0.56	0.11	19.15
IFPM	211	10.158	1.09	4.19	1.15	27.54
IFLM	211	16.38	3.39	7.48	2.15	28.75

^a^ CW: carcass weight; LVW: liver weight; AFW: abdominal fat weight; HEW: heart weight; LVR: liver to body ratio; AFP abdominal fat percentage; HER: heart to body ratio; IFPM: intramuscular fat of pectoral muscle; IFLM: intramuscular fat of leg muscle.

**Table 2:** The phenotypic above diagonal and genetic below diagonal correlation between nine carcass traits.

	CW	LVW	AFW	HEW	LVR	AFP	HER	IFPM	IFLM
CW	1	0.281 **	0.341 **	0.701 **	−0.402 **	0.264 **	−0.031	−0.097	0.085
LVW		1	−0.126	0.347 **	0.743 **	−0.167 *	0.207 **	0.169 *	0.007
AFW			1	−0.026	−0.331 **	0.989 **	−0.337 **	0.093	0.276 **
HEW				1	−0.133	−0.083	0.875 **	−0.064	0.039
LVR					1	−0.329 **	0.240 **	0.242 **	−0.047
AFP						1	−0.345 **	0.102	0.275 **
HER							1	0.008	−0.011
IFPM								1	0.157 *
IFLM									1

CW: carcass weight; LVW: liver weight; AFW: abdominal fat weight; HEW: heart weight; LVR: liver to body ratio; AFP abdominal fat percentage; HER: heart to body ratio; IFPM: intramuscular fat of pectoral muscle; IFLM: intramuscular fat of leg muscle. ** *p* < 0.01, and * *p* < 0.05.

**Table 3:** Significant SNPs and candidate genes associated with AFW, AFP, IFPM, and IFLM traits.

Traits	Chr	nSNP	Position (Mb)	nGene	Effect ^a^	Gene
AFW	1	1	109,198,052	4	14.75	*PRDM15*, *C2CD2*, *ZBTB21*, *UMODL*
	2	1	51,692,642	3	11.83	*BLVRA*, *VOPP1*, *LANCL2*
	2	1	66,761,782	1	−7.05	*GMDS*
	10	1	66,761,782	16	−12.54	*SCAMP2*, *ULK3*, *CPLX3*, *CSK*, *CYP1A2*, *CYP1A1*, *EDC3*, *CLK3*, *ARID3B*, *ACTG1L*, *UBL7*, *SEMA7A*, *CYP11A1*, *STRA6*, *ISLR*, *ISLR2*
	13	1	2,535,341	3	26.69	*FAM114A2*, *GRIA1*, *MFAP3*
	35	1	11,945,109	1	30.32	*PA28_beta*
AFP	1	1	112,969,661	3	0.87	*OTC*, *TSPAN7*, *RPGR*
	7	1	5,897,982	4	0.76	*TRAF3IP1*, *USP40*, *UGT1A1*, *SH3BP4*
	9	1	21,433,493	-	−0.63	*-*
	10	1	13,199,669	6	−1.75	*ACAN*, *AEN*, *DET1*, *RPS11*, *NUDIX*, *NTRK3*
	13	1	11,945,109	3	1.93	*FAM114A2*, *GRIA1*, *MFAP3*
IFLM	1	1	149,287,395	-	2.76	*-*
	2	1	79,956,861	16	−1.31	*OTC*, *RPGR*, *NUDIX*, *NTRK3*, *UGT1A1*, *TSPAN7*, *MFAP3*, *ACAN*, *AEN, DET1*, *FAM114A2*, *SH3BP4,* *RPS11*, *USP40*, *TRAF3IP1*, *NUDIX*
	2	1	144,069,823	1	1.33	*KCNK9*, *TRAPPC9*
	4	1	89,896,431	-	0.78	*-*
	5	1	22,865,617	3	2.35	*CKAP5*, *LRP4*, *C11orf49*
	5	1	28,039,569	1	0.98	*RAD51B*
	25	1	22,865,617	20	0.66	*UBE2Q1*, *CHRNB2*, *ADAR*, *KCNN3*, *PBXIP1*, *PYGO2,* *SHC1*, *CKS1B*, *FLAD1*, *LOC112530287*, *ZBTB7B*, *HCN3*, *KHDC4*, *DCST2*, *LOC107050229*, *LOC107049672*, *FDPS*, *SCAMP3*, *CLK2*, *ASH1L*
IFPM	1	1	25,946,497	-	1.18	-
	1	1	135,236,335	4	1.03	*MRPS9*, *TGFBRAP1*, *C2orf49*, *FHL2*
	6	1	29,803,881	4	1.33	*VAX1*, *KCNK18*, *HSPA12A*, *SHTN1*
	9	1	22,557,616	6	1.38	*VEPH1*, *GMPS*, *LEKR1*, *TIPARP*, *SSR3*, *KCNAB1*
	12	1	13,972,290	3	0.63	*PRICKLE2*, *ADAMTS9*, *CCNL1*
	23	1	1,059,926	3	0.28	*ZCCHC17*, *FABP3*, *SERINC2*
	26	1	3,635,010	9	0.24	*LRIG2*, *MAGI3*, *TAFA3*, *WNT2B, ST7L*, *CAPZA1*, *MOV10*, *RHOC*, *PPM1J*
	28	1	1,544,929	15	1.10	*UNC13A*, *MYO5B, PLPP2*, *LOC100857637*, *NFIC*, *FZR1,* *LOC100858505*, *PIP5K1C*, *TBXA2R*, *HMG20B*, *DOHH, MFSD12*, *LOC101748203*, *C19orf71*, *CACTIN*

^a^ Positive values indicated an increase in the trait value of the effect allele; negative values indicated a decrease in the trait value of the effect allele.

**Table 4:** KEGG pathway enrichment analysis for candidate genes of AFW, AFP, IFPM, and IFLM.

Traits	ID	Description	*p*-Value	Key Genes
AFW	ko00232	Caffeine metabolism	4.29 × 10^4^	*CYP1A2*, *CYP1A1*
	ko00140	Steroid hormone biosynthesis	1.19 × 10^3^	*CYP1A2*, *CYP1A1*, *CYP11A1*
	ko04913	Ovarian steroidogenesis	1.87 × 10^3^	*CYP1A2*, *CYP1A1*, *CYP11A1*
	ko00591	Linoleic acid metabolism	8.05 × 10^3^	*CYP1A2*, *CYP1A1*
	ko00830	Retinol metabolism	1.32 × 10^2^	*CYP1A2*, *CYP1A1*
	ko00980	Metabolism of xenobiotics by cytochrome P450	1.33 × 10^2^	*CYP1A2*, *CYP1A1*
	ko00982	Drug metabolism-cytochrome P450	1.33 × 10^2^	*CYP1A2*, *CYP1A1*
	ko00380	Tryptophan metabolism	1.57 × 10^2^	*CYP1A2*, *CYP1A1*
	ko05204	Chemical carcinogenesis-DNA adducts	2.03 × 10^2^	*CYP1A2*, *CYP1A1*
AFP	ko00220	Arginine biosynthesis	1.66 × 10^2^	*OTC*
	ko00053	Ascorbate and aldarate metabolism	1.76 × 10^2^	*UGT1A1*
	ko00040	Pentose and glucuronate interconversions	2.17 × 10^2^	*UGT1A1*
	ko00860	Porphyrin metabolism	2.47 × 10^2^	*UGT1A1*
	ko05033	Nicotine addiction	4.19 × 10^2^	*GRIA1*
	ko00830	Retinol metabolism	4.49 × 10^2^	*UGT1A1*
IJPM	ko04011	MAPK signaling pathway-yeast	6.72 × 10^3^	*RHOC*, *PIP5K1C*
	ko04310	Wnt signaling pathway	9.21 × 10^3^	*PRICKLE2*, *WNT2B*, *RHOC*
	ko04072	Phospholipase D signaling pathway	1.36 × 10^2^	*PLPP2*, *RHOC*, *PIP5K1C*
	ko04144	Endocytosis	4.12 × 10^2^	*CAPZA1*, *RHOC*, *PIP5K1C*
	ko04666	Fc gamma R-mediated phagocytosis	4.24 × 10^2^	*PLPP2*, *PIP5K1C*
	ko05231	Choline metabolism in cancer	431 × 10^2^	*PLPP2*, *PIP5K1C*
IJLM	ko00511	Other glycan degradation	2.631 × 10^5^	*LOC107050229*, *SCAMP3*
	ko00600	Sphingolipid metabolism	4.01 × 10^4^	*LOC107050229*, *SCAMP3*
	ko00900	Terpenoid backbone biosynthesis	1.35 × 10^3^	*FDPS*

## Data Availability

The raw data supporting the conclusions of this article will be made available by the authors on request.

## Supplementary Materials

The following supporting information can be downloaded at: https://www.mdpi.com/article/10.3390/ani15223342/s1, Table S1. The main nutritional components of feed.
