# Supplementary material for: Genome-Wide Association Study of Abdominal and Intramuscular Fat Deposition Traits in Huainan Yellow-Feathered Chickens
Source: Animals (Basel). 2025 Nov 19;15(22):3342. doi: 10.3390/ani15223342 (PMC12649552; doi:10.3390/ani15223342)
Supplement: Supplementary file 1 [file animals-15-03342-s001.zip › animals-3986953-supplementary.pdf]

Supplementary Table S1. The main nutritional components of feed

| Ingredients | Value |
|---|---|
| Crude protein（g/100g） | 20% |
| Crude fat（g/kg） | 75 |
| Crude ash content（%） | 8.3 |
| water content（g/100g） | 10.7 |
| Crude fiber（%） | 4.8 |
| Calcium（%） | 1.61 |
| Total phosphorus（%） | 0.55 |
| Metabolic energy（MJ/kg） | 12.54 |
